# *In vitro* and *in silico* biopotentials of phytochemical compositions and antistaphylococcal and antipseudomonal activities of volatile compounds of *Argania spinosa* (L.) seed oil

**DOI:** 10.3389/fbioe.2024.1348344

**Published:** 2024-03-13

**Authors:** Mohammed S. Almuhayawi, Mohammed H. Alruhaili, Hattan S. Gattan, Mohanned Talal Alharbi, Mohammed K. Nagshabandi, Nashwa Hagagy, Saad M. Almuhayawi, Soad K. Al Jaouni, Samy Selim, Ehab M. Mostafa, Mohamed E. Elnosary

**Affiliations:** ^1^ Department of Clinical Microbiology and Immunology, Faculty of Medicine, King Abdulaziz University, Jeddah, Saudi Arabia; ^2^ Special Infectious Agents Unit, King Fahad Medical Research Center, King AbdulAziz University, Jeddah, Saudi Arabia; ^3^ Department of Medical Laboratory Sciences, Faculty of Applied Medical Sciences, King Abdulaziz University, Jeddah, Saudi Arabia; ^4^ Department of Medical Microbiology and Parasitology, Faculty of Medicine, University of Jeddah, Jeddah, Saudi Arabia; ^5^ Department of Biology, College of Science and Arts at Khulis, University of Jeddah, Jeddah, Saudi Arabia; ^6^ Department of Otolaryngology-Head and Neck Surgery, Faculty of Medicine, King Abdulaziz University, Jeddah, Saudi Arabia; ^7^ Botany and Microbiology Department, Faculty of Science, Suez Canal University, Ismailia, Egypt; ^8^ Department of Hematology/Oncology, Yousef Abdulatif Jameel Scientific Chair of Prophetic Medicine Application, Faculty of Medicine, King Abdulaziz University, Jeddah, Saudi Arabia; ^9^ Department of Clinical Laboratory Sciences, College of Applied Medical Sciences, Jouf University, Sakaka, Saudi Arabia; ^10^ Department of Pharmacognosy, College of Pharmacy, Jouf University, Sakaka, Saudi Arabia; ^11^ Pharmacognosy and Medicinal Plants Department, Faculty of Pharmacy (Boys), Al-Azhar University, Cairo, Egypt; ^12^ Botany and Microbiology Department, Faculty of Science, Al-Azhar University, Nasr City, Cairo, Egypt

**Keywords:** bacterial infections, *Staphylococcus aureus*, *Pseudomonas aeruginosa*, argan seed essential oil, biopotentials, antibacterial activity

## Abstract

Active components in medicinal plants provide unlimited useful and traditional medicines. Antimicrobial activities are found in secondary metabolites in plant extracts such as argan oil. This experimental investigation aims to determine argan oil’s volatile compounds and examine their *in vitro* antimicrobial properties. *In silico* simulations, molecular docking, pharmacokinetics, and drug-likeness prediction revealed the processes underlying the *in vitro* biological possessions. Gas chromatography–mass spectrometry (GC/MS) was used to screen argan oil’s primary components. *In silico* molecular docking studies were used to investigate the ability of the selected bioactive constituents of argan oil to act effectively against *Pseudomonas aeruginosa* and *Staphylococcus aureus* (*S. aureus*) isolated from infections. The goal was to study their ability to interact with both bacteria’s essential therapeutic target protein. The 21 chemicals in argan oil were identified by GC/MS. Docking results for all compounds with *S. aureus* and *P. aeruginosa* protease proteins ranged from −5 to −9.4 kcal/mol and −5.7 to −9.7 kcal/mol, respectively, compared to reference ligands. Our docking result indicates that the 10-octadecenoic acid, methyl ester was the most significant compound with affinity scores of −9.4 and −9.7 kcal/mol for *S. aureus* and *P. aeruginosa* proteins, respectively. The minimal bactericidal concentration (MBC) and minimal inhibitory concentration (MIC) of argan oil were 0.7 ± 0.03 and 0.5 ± 0.01 for *S. aureus* and 0.4 ± 0.01 and 0.3 ± 0.02 for *P. aeruginosa*, respectively. We confirmed the antimicrobial properties of argan oil that showed significant growth inhibition for *S. aureus* and *P. aeruginosa.*

## 1 Introduction

*Argania spinosa* (L.), an indigenous tree of southern Morocco, is a member of the Sapotaceae family (El Monfalouti et al., 2010; Hebi and Khallouki, 2022). *Argania spinosa* (L.) press-cake is a rich source of diverse bioactive phytochemicals when it comes to the bioactive elements of the *Argania spinosa* (L.) tree (phenolic compounds) (El Monfalouti et al., 2012) and is the bioproduct of the production of argan oil (Khallouki et al., 2003).

The composition of argan oil is as follows: 0.5% linolenic, 6% stearic, 12% palmitic, and 42.8% oleic acids. Phenols, fatty acids, carotenes, tocopherols (vitamin E), and squalene are all present in argan oil (80% unsaturated fatty acids). Catechin, oleuropein, epicatechin, tyrosol, resorcinol, catechol, vanillic acid, and caffeic acid are the primary natural phenols in argan oil. Argan oil may be more oxidation-resistant than olive oil, depending on the extraction process (Charrouf and Guillaume, 2007).

The argan seeds that are pressed to extract the oil merit special consideration (Lakram et al., 2019). Even though argan seed cake has primarily been used for animal feed (Cherki et al., 2006), it has also been suggested as a natural moisturizer and exfoliator for use in commercial shampoos and lotions to heal wounds, sprains, and scabies topically. Recent studies show that the ethanol extract of argan seed cake may be utilized as a natural lightening and pharmacological therapy for treating hyperpigmentation issues because of its effects on melanogenesis (Bourhim et al., 2018; Bourhim et al., 2021; ZeinEldin et al., 2023).

According to several studies, the ingredients in argan press-cake exhibit anti-inflammatory, antibacterial, antifungal, free-radical scavenging, and depigmenting activities (Charrouf and Guillaume, 2009; Elshama, 2018; Koufan et al., 2020; Al-Mokadem et al., 2022; Gharby and Charrouf, 2022). Plant extracts, medicinal oils, and essential oils can all be used for various purposes. The antioxidant, insecticidal, antifungal, anticancer, antiviral, and antibacterial effects of essential oils have been investigated (Sylvestre et al., 2006; Elshazly et al., 2023). The existence of two plant phenolic chemicals, carvacrol and thymol, which are primarily responsible for all plant oils’ antibacterial activity, has been demonstrated (Chao et al., 2000).

*S. aureus* is a spherical-shaped, Gram-positive, non-motile, and non-spore-forming microorganism. In 1880, Alexander Ogston isolated the first strain of bacteria while researching septicemia and wound infection bacteria. Gram-positive cocci were found in 88 pus samples after being examined under a microscope (Licitra, 2013). *S. aureus* is a historically significant emerging zoonotic pathogen with veterinary and public health implications. *S. aureus* can withstand unfavorable environmental conditions such as desiccation and sunlight, creating significant issues in humans and animals (Atoum et al., 2003; Weese and van Duijkeren, 2010). The bacterium can attack internal organs, mucous membranes, and the skin, causing severe diseases in animals and humans, including suppurative skin infections, respiratory tract infections, osteomyelitis, septicemia, endocarditis, and acne (Alaklobi et al., 2015). In addition, *S. aureus* is one of the principal causes of cattle mastitis (Elsayed and Dawoud, 2015).

*P*. *aeruginosa* is an important pathogen to animals and humans, but it is seldom involved in primary infections. *P. aeruginosa* is a significant nosocomial pathogen that primarily causes hospital-acquired pneumonia in immunocompromised people. It has been thought to be the specific cause of infections in animals, particularly in dogs, including wound/urinary tract infections, superficial skin infections, perianal abscesses, chronic deep pyoderma, and otitis externa (Hillier et al., 2006; Bloom, 2014). *P. aeruginosa* infections are common (11.5% in Europe and 17% in developed countries) (Bloom, 2014). *P. aeruginosa* expresses antibiotic resistance that can be acquired through plasmids or transposons or naturally. Due to its high colonization potential, ability to ruin food, and resistance to antibiotics, disinfectants, and antiseptics, *P. aeruginosa* is frequently associated with biofilm formation nosocomial infections and food poisoning (Morris, 2004; Hillier et al., 2006). Otitis media (OM) is the medical name for an infection of the tympanic membrane or middle ear, characterized by ear pain or discharge (Danishyar and Ashurst, 2017; Sander, 2001; Kono et al., 2021).

According to our knowledge, the antibacterial (antistaphylococcal and antipseudomonal) activity of argan oil has been investigated by few studies till now. Therefore, the current study was designed and suggested to establish the effect of argan oil on the multidrug-resistant *S. aureus* and *P. aeruginosa* isolated from patients. Also, *in silico* simulations, including molecular docking, pharmacokinetics (ADMET), and drug-likeness expectation, were used to expose the practices essential to the *in vitro* antimicrobial possessions.

## 2 Results

### 2.1 Characterization of the isolated compounds

The chemical makeup of the essential oil of *Argania spinosa* is provided in Table 1, with the components listed according to their elution order on the TG-5MS column, while the total ion chromatogram of argan essential oil, shown in Supplementary Figure S1, displays the mass spectra of the investigated compounds at 70 eV, and Table 1 lists the principal fragment ions and their relative intensities (Rt).

**TABLE 1:** Chemical composition of the main components of argan oil.

No.	Identified compounds	R_t_ (min)	Area (%)	Molecular	Molecular
				Formula	Weight
1	Butylated hydroxytoluene	16.47	7.9	C15H24O	220
2	Octanal, 2-(phenylmethylene)-	21.44	3.34	C15H20O	216
3	Isopropyl myristate	23.62	8.81	C17H34O2	270
4	Palmitic acid, methyl ester	25.52	2.45	C17H34O2	270
5	Hexadecanoic acid	26.18	6.89	C16H32O2	256
6	1-Heptatriacotanol	26.57	1.26	C37H76O	536
7	14-á-H-pregna	28.04	2.98	C21H36	288
8	7,10-Hexadecanoic acid, methyl ester	28.52	2.46	C19H34O2	294
9	10-Octadecenoic acid, methyl ester	28.71	9.2	C19H36O2	296
10	9,12-Octadecadienoic acid (Z,Z)-	29.19	8.26	C19H34O2	280
11	Oleic acid	29.38	14.11	C18H34O2	282
12	Ethyl oleate	29.92	8.39	C20H38O2	310
13	Tetrapentacontane, 1,54-dibromo-	31.04	1.58	C54H108Br2	914
14	14-á-H-pregna	31.52	3.56	C21H36	288
15	14-á-H-pregna	32.7	2.07	C21H36	288
16	17-Pentatriacontene	33.15	2.55	C35H70	490
17	Tetrapentacontane, 1,54-dibromo-	34.31	2.45	C54H108Br2	914
18	17-Pentatriacontene	34.72	3.86	C35H70	490
19	Tetrapentacontane, 1,54-dibromo-	35.85	2.02	C54H108Br2	914
20	17-Pentatriacontene	37.72	2.87	C35H70	490
21	1-Heptatriacotanol	40.06	3.01	C37H76O	536

The identification of the chemical ingredients was given based on a comparison of their retention indices and mass spectra in the system’s database. The reactivity and chemistry of medicinal plants such as Argania (*Argania spinosa*) have always been of good interest because of their essential role in the treatment of several diseases.

### 2.2 Molecular modeling study

#### 2.2.1 Molecular docking study

**FIGURE 1:**
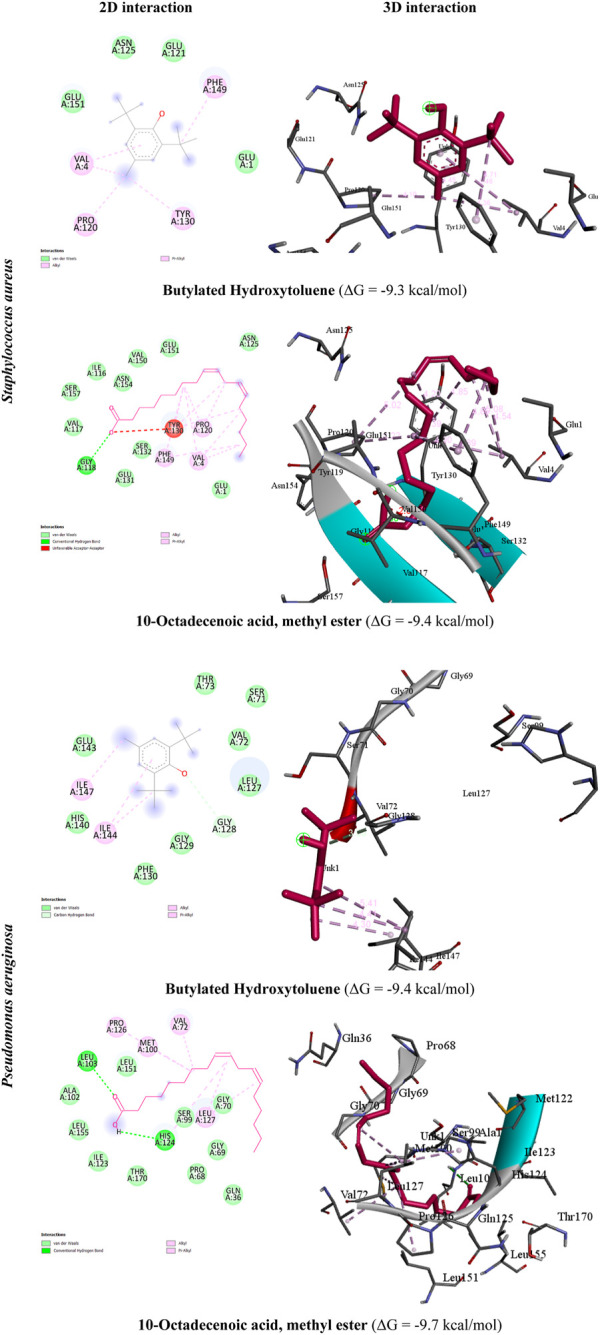
2D and 3D interaction patterns of top-scoring compounds in the protease protein of *Staphylococcus aureus* and *Pseudomonas aeruginosa* binding active sites while showing non-covalent bond distances.

In this study, we focused on estimating the binding affinity in Kcal/mol for our isolated compounds against the protease protein of *Staphylococcus aureus* (PDB Id: 5MM8) and protease protein of *Pseudomonas aeruginosa* (PDB Id: 7M1M). This helped our approach in interpreting the obtained biological activity for the tested extracts for future structural optimization to obtain more potent analogs. The X-ray crystal structure against the protease protein of *Staphylococcus aureus* (PDB Id: 5MM8) and the protease protein of *Pseudomonas aeruginosa* (PDB Id: 7M1M) was downloaded from the Protein Data Bank (PDB) database (www.rcsb.org). The protease protein in *S. aureus* and *P. aeruginosa* is central to bacterial proteolysis and controls cellular physiology and stress responses. A rigid and accurate docking type was used for each docking experiment, and the results were calculated based on 250 different sequential runs. The docking result values were obtained regarding energy full fitness and delta G Kcal/mol. Those binding models possessing the most favorable energies (lowest delta G Kcal/mol) were estimated by the fast analytical continuum treatment of solvation. The average full fitness of the contributing elements was used to cluster binding modes. The range of the argan oil compound’s molecular docking binding affinity score against the protease protein of the *Pseudomonas aeruginosa* (PDB Id: 7M1M) target was −5 to −9.4 kcal/mol. The best complex, however, is 10-octadecenoic acid, methyl ester since it has a low energy value of −9.4 kcal/mol, followed by butylated hydroxytoluene, which has an energy value of −9.3 kcal/mol, while the range of the argan oil compound’s molecular docking binding affinity score against the protease protein of the *Staphylococcus aureus* (PDB Id: 5MM8) target was −5.6 to −9.7 kcal/mol. The best complex, however, is 10-octadecenoic acid, methyl ester since it has a low energy value of −9.7 kcal/mol, followed by butylated hydroxytoluene, which has an energy value of −8.9 kcal/mol. On the other hand, the molecule with the lowest energy is believed to be the good one. The interaction patterns and visualization of the resulting docking poses were accomplished offline using Discovery Studio 2021 (BIOVIA/Discovery Studio v1.0.20298) Figure 1. Validation for our docking protocol was carried out via re-docking of the co-crystallized ligand. We discovered that the EA-dock workflow implemented in the SwissDock server (http://www.swissdock.ch) could re-produce a docking pose of the co-crystallized ligand within 2 Å RMSD from coordinates in both protein structures (PDB Id: 5MM8 and 7M1M). A summary of the estimated binding energy with the residues of the protease protein of *Staphylococcus aureus* (PDB Id: 5MM8) and the protease protein of *Pseudomonas aeruginosa* (PDB Id: 7M1M) of the isolated compounds is shown in Table 2.

**TABLE 2:** Estimated binding energy of isolated compounds in the protease protein of *Staphylococcus aureus* (PDB Id: 5MM8) and *Pseudomonas aeruginosa* (PDB Id: 7M1M) binding active sites.

Entry no.	Compounds name	Estimated ΔG (kcal/mol)	Estimated ΔG (kcal/mol)
		(PDB Id: 5MM8)	(PDB Id: 7M1M)
1	Acetate ion (PDB Id: 5MM8; referenced ligand)	−6.4	—
2	(4S)-2-Methyl-2,4-pentanediol	—	−5.9
	(PDB Id: 7M1M; referenced ligand)		
3	Butylated hydroxytoluene	−9.3	−8.9
4	Octanal, 2-(phenylmethylene)-	−7.4	−7.1
5	Isopropyl myristate	−7.5	−6
6	Palmitic acid, methyl ester	−7.2	−5.7
7	Hexadecanoic acid	−7.8	−6.1
8	1-Heptatriacotanol	−6.6	−5.7
9	7,10-Hexadecanoic acid, methyl ester	−7.7	−6.4
10	10-Octadecenoic acid, methyl ester	−9.4	−9.7
11	9,12-Octadecadienoic acid (Z,Z)-	−8.2	−6.9
12	Oleic acid	−8.2	−6.1
13	Ethyl oleate	−8.4	−7.6
14	Tetrapentacontane, 1,54-dibromo-	−5	−5.6
15	17-Pentatriacontene	−7.2	−6.5

#### 2.2.2 Evaluation of the drug-likeness, drug score, and toxicity risks of the isolated compounds

The calculated properties of the drug-likeness, toxicity risks, and drug score of the isolated compounds are shown in Table 3. The log *p-value* for a compound (log(_octanol_/_water_)) provides a reliable estimate of hydrophilicity for the compound series. Low hydrophilicity from a high log *p*-value could translate into poor drug absorption or permeation. A compound with a log *p*-value not exceeding 5.0 will likely be absorbed efficiently. We can see from Table 3 that our isolated compounds (entries 1–2) are very lipophilic with a log *p*-value of more than 5. Aqueous solubility, indicated by LogS, is another factor provided by OSIRIS Property Explorer that affects drug absorption and distribution. Impaired absorption of compounds could be a result of poor water solubility. The OSIRIS program represents LogS values in mol/liter. The log value for 80% of the traded drugs was found to be more than −4. The results in Table 3 show that entries 1–2 have a LogS value greater than −4. For molecular weights, the best approach is to keep them as low as possible (less than 500) to achieve good absorption and delivery to the site of action. All the isolated compounds maintain low molecular weight except 1-heptatriacotanol (entry 6), 7,10-hexadecanoic acid, methyl ester (entry 7), and tetrapentacontane, 1,54-dibromo- (entry 12). The total polar surface area (TPSA) is calculated by summating the polar moieties of a molecule’s surface. It correlates well with different bioavailability properties, such as intestinal absorption and the ability to penetrate the blood–brain barrier. If the polar atoms exposed to the molecule’s surface sum up to around 80 or 100 Å^2^, the molecules will likely pass the membranes easily. Molecules with a TPSA value of more than 140 Å^2^ are more likely to have problems with oral bioavailability like 7,10-hexadecanoic acid and methyl ester (entry 7). The other isolated compounds are more likely to have no issues regarding bioavailability following oral administration.

**TABLE 3:** Calculated ADME properties, drug-likeness, drug score, and toxicity risk of the isolated compounds.

Entry	Comp. no./ID	cLogP	LogS	Mol weight	TPSA (Å^2^)	Drug-likeness	Drug score	Toxicity risks (mutagenicity, tumorigenicity, irritancy, and reproductive effects)
1	Butylated hydroxytoluene	4.24	−3.27	220.35	20.23	19.56	0.84	None
2	Octanal, 2-(phenylmethylene)-	4.03	−3.85	21.32	17.07	6.47	0.47	None
3	Isopropyl myristate	5.53	−7.59	270.45	26.30	−4.53	0.21	None
4	Palmitic acid, methyl ester	5.53	−6.01	270.45	26.30	−3.42	0.11	None
5	Hexadecanoic acid	5.2	−5.31	250.42	37.3	−1.24	0.17	None
6	1-Heptatriacotanol	13.02	−19.16	537	20.23	−2.27	0.27	Tumorigenic
7	7,10-Hexadecanoic acid, methyl ester	10.1	−16.58	1130	190.20	−7.96	−0.23	Mutagenic, irritant, and reproductive effects
8	10-Octadecenoic acid, methyl ester	6.04	−8.18	310.51	26.30	0.92	0.81	None
9	9,12-Octadecadienoic acid (Z,Z)-	5.45	−7.58	280.45	37.30	−1.12	0.16	Mutagenic
10	Oleic acid	5.71	−8.26	282.46	37.30	−16.34	0.21	
11	Ethyl oleate	6.33	−8.44	310.51	26.30	−1.74	0.24	Mutagenic
12	Tetrapentacontane, 1,54-dibromo-	20.48	−29.82	917.24	0	−2.58	0.09	Irritant effects
13	17-Pentatriacontene	13.13	−18.48	490.93	0	−3.47	0.36	None

Yellow-colored cells are those violating the permissible limits.

OSIRIS software Explorer uses fragment-based drug-likeness, calculated based on the drug-likeness scores of 5,300 diverse substructure fragments. The fragment library was established by collecting subunits from 3,300 marketed drugs and 15K commercial chemical substances (Fluka). A statistical analysis of drug-likeness values shows that nearly 80% of drug molecules lie in the positive range, while most Fluka chemicals exist in the negative range. As a result, it is a good idea to maintain the drug-likeness value of drug candidates in the positive range. We could see that only entries 1, 2, and 8 show a drug-likeness value in the positive range. Toxicity risk alerts generated by the OSIRIS program indicate that the supplied structure may show dangerous effects regarding specific risk categories. The prediction process depends on whether the submitted compounds contain a structural fragment that produces toxicity alerts.

The results were mainly based on the assumption that drugs are mostly toxicity-free. Risky fragments represent a substructure of harmful compounds that never or rarely show in marketed drugs. One thing to remember is that the presence or absence of risk alerts means that the submitted compound will or will not yield any toxic effects. We could see that entries 6, 7, and 9–12 showed potential toxicity risks, while the rest of the isolated compounds did not show any tendency to produce toxic effects. Drug score is usually calculated using cLogP, LogS, molecular weight, drug-likeness, and toxicity risks in one value. This one-handy parameter could be used to determine if the compound would be eligible to be a drug molecule. Entries 1, 28, and 13 exhibit a good drug score of more than 0.35. Butylated hydroxytoluene (entry 1) and 10-octadecenoic acid, methyl ester (entry 8) showed good docking scores with the molecular targets, no potential toxicity risks, and drug scores of more than 0.8. This indicates that the two compounds have high potential for future development as drug candidates.

#### 2.2.3 Evaluation of the pharmacokinetics, cellular permeability, and medicinal chemistry friendliness of the isolated compounds

The pharmacokinetics, ADME, drug-likeness, and medicinal chemistry friendliness for isolated compounds were calculated with an accessible online web server (SwissADME), and they are shown in Table 4. The BOILED-Egg model (Daina and Zoete, 2016) determined passive permeation in gastrointestinal absorption and through the blood–brain barrier (BBB). The isolated phytochemical compounds have low GIT permeability except for isopropyl myristate, palmitic acid methyl ester, hexadecanoic acid, 10-octadecenoic acid, methyl ester, 9,12-octadecadienoic acid (Z, Z)-, and oleic acid. In addition, isopropyl myristate, palmitic acid methyl ester, hexadecanoic acid, and 9,12-octadecadienoic acid (Z, Z)- are the only analogs that exhibited high BBB permeability between the isolated phytochemicals (Table 4). Drug-likeness value could indicate a drug molecule’s possibility of becoming an orally administered candidate with acceptable bioavailability. All the isolated phytochemical compounds showed 0–2 violations except to Lipinski’s rule of five (Lipinski et al., 1997) except for 7,10-hexadecanoic acid, methyl ester.

**TABLE 4:** Calculated ADME properties of the isolated compounds.

Entry	Comp. no./ID	GI absorption	BBB permeant	Lipinski^e^	PAINS^f^ N. alert	Bioavailability Score	Synthetic accessibility
				N. violations			
1	Butylated hydroxytoluene	Low	No	0	0	0.55	1.48
2	Octanal, 2-(phenylmethylene)-	Low	No	0	0	0.55	2.53
3	Isopropyl myristate	High	Yes	1	0	0.55	2.68
4	Palmitic acid, methyl ester	High	Yes	1	0	0.55	2.53
5	Hexadecanoic acid	High	Yes	1	0	0.85	2.31
6	1-Heptatriacotanol	Low	No	2	0	0.17	4.87
7	7,10-Hexadecanoic acid, methyl ester	Low	No	3	0	0.11	9.24
8	10-Octadecenoic acid, methyl ester	High	No	1	0	0.55	3.73
9	9,12-Octadecadienoic acid (Z,Z)-	High	Yes	1	0	0.85	3.10
10	Oleic acid	High	No	1	0	0.85	3.07
11	Ethyl oleate	Low	No	1	0	0.55	3.34
12	Tetrapentacontane, 1,54-dibromo-	Low	No	2	0	0.17	9.07
13	17-Pentatriacontene	Low	No	1	0	0.55	5.57

Medicinal chemistry friendliness is an additional term medicinal chemists use to guide their endeavors to develop new chemical entities. A primary and essential step in any drug discovery project is the ability to recognize any possibly problematic fragments/patterns and then decide to prioritize the compounds to be pursued. PAINS (pan assay interference compounds, frequent hitters, and promiscuous compounds) are molecules containing substructure moieties showing potent responses in multiple assays irrespective of the target protein. If those fragments lead to false positives in six orthogonal assays, they would be labeled a potential threat to furnish frequent hitters (Baell and Holloway, 2010). The SwissADME server would raise red flags if such substructures were detected in the submitted molecule. All isolated phytochemical compounds showed zero alerts for the PAINS filter.

The Abbot Bioavailability Score is based on paying close attention to calculating compound chances to have at least 10% oral bioavailability in a rat or Caco-2 permeability model. This bioavailability score depends on the total charge, TPSA, and violation of the Lipinski rule of five. This score would also help to classify submitted compounds into four different probabilities of 11%, 17%, 56%, or 85%. All our isolated phytochemical compounds scored either 0.17 or 0.55, except for hexadecanoic acid, which scored 0.85. Synthetic accessibility (SA) is another factor provided by the SwissADME server, which helps medicinal chemists decide if submitted molecules could be synthesized easily and tested in biological assays. It ranges from 1 (very easy) to 10 (very difficult). The synthetic accessibility range was derived from the contribution of 1,024 building blocks depending on their size and complexity. All the isolated phytochemical compounds have various SA scores ranging from 1.48 to 9.07 (Fukunishi et al., 2014).

### 2.3 Antibacterial activity

The efficacy of argan oil against the inhibition and growth reduction of *S. aureus* and *P. aeruginosa* bacteria was investigated. Argan essential oil showed a higher effect on *S. aureus* and *P. aeruginosa* bacteria when compared with negative (methanol 50%) and positive (referenced antibiotics) controls. Except for the chloramphenicol antibiotic, argan essential oil had more effects on *S. aureus* and *P. aeruginosa* than the referenced antibiotics used in treating bacterial infections. The present study illustrated that the antibacterial activity was shown only in the lipophilic phase, whereas the aqueous extract did not cause effects.

The results revealed a considerable inhibitory capacity of argan oil toward *S. aureus* and *P. aeruginosa* activity with inhibition zone diameters of 28 ± 1.5 and 24 ± 0.9 mm at a concentration of 0.5 and 0.3 mg/mL, respectively, and it is highly significant when compared to the negative control and *p*-value <0.05 (Table 5).

**TABLE 5:** Inhibition zone, MIC, MBC, and tolerance ratio of argan essential oil on *S. aureus* and *P. aeruginosa*.

Bacterial species	Inhibition zone diameter (mm)	MIC (mg/mL)	MBC (mg/mL)	Tolerance ratio (MBC/MIC)
Negative control	0	0	0	0
*Staphylococcus aureus*	28 ± 1.5**	0.5 ± 0.01	0.7 ± 0.03	1.4 ± 0.1
*Pseudomonas aeruginosa*	24 ± 0.9**	0.3 ± 0.02	0.4 ± 0.01	1.3 ± 0.09

MIC, minimum inhibitory concentration; MBC, minimum bactericidal concentration; mean ± SD and **highly significant with *p*-value <0.05.

Minimal inhibitory concentration (MIC) and minimal bactericidal concentration (MBC) tests are used often to determine the activity of the drug on certain species of bacteria. The MBC is complementary to the MIC; while the MIC test demonstrates the lowest level of antimicrobial agent that greatly inhibits growth, the MBC test demonstrates the lowest level of antimicrobial agent resulting in microbial death. If the ratio of the MBC to MIC is small (less than 4–6), a drug is considered bactericidal, and it is possible to obtain drug concentrations that will kill 99.9% of the organisms exposed.

The MBC and MIC (mg/mL) of argan essential oil against *S. aureus* and *P. aeruginosa* are shown in Table 5. The MIC test showed that all potent investigated argan essential oils inhibited *P. aeruginosa* and *S. aureus* at a concentration above 0.3 mg/mL and 0.5 mg/mL, respectively, while the MBC was equal to 0.7 mg/mL for *Staphylococcus aureus* and 0.4 mg/mL for *P. aeruginosa* (Table 5).

#### 2.3.1 Scanning electron microscopy

**FIGURE 2:**
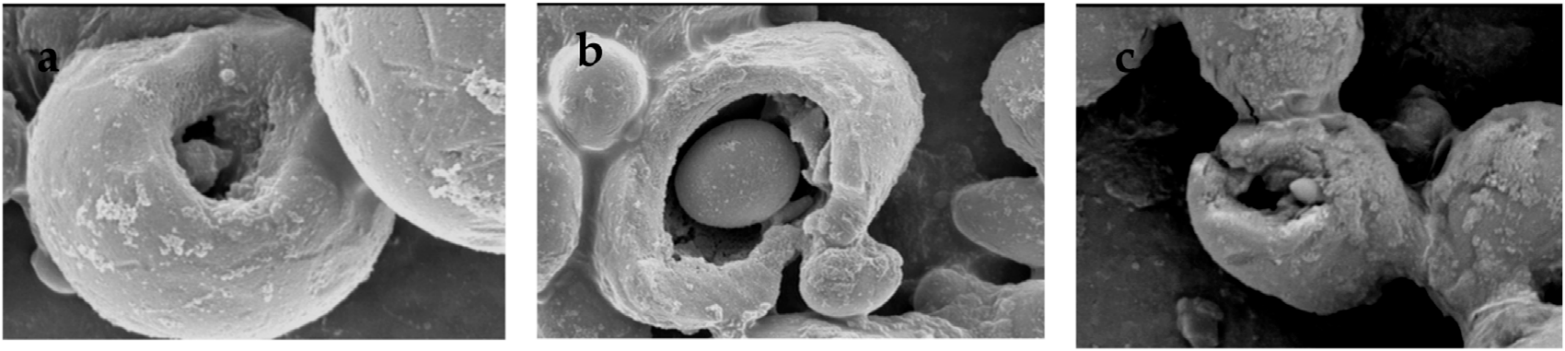
Scanning electron microscopy images of the antibacterial effect of argan oil against **(A, B)** *Staphylococcus aureus* and **(C)** *Pseudomonas aeruginosa*.

The scanning electron microscopy (SEM) analysis showed that the incubation of argan oil with *S. aureus* (Figures 2A, B) and *P. aeruginosa* (Figure 2C) caused some cells to be deformed and some cells to be damaged.

## 3 Discussion

The central precepts of utilizing argan oil as an antibacterial agent are that it is a safe, affordable, and readily accessible herbal oil with significant medicinal significance. Because there is little to no resistance and fewer harmful side effects from taking conventional antibiotics, there is a clear need to use medicinal or herbal oils.

Using gas chromatography–mass spectrometry (GC/MS), the chemical composition of argan oil was examined. Compounds such as butylated hydroxytoluene, oleic acid, ethyl oleate, 10-octadecenoic acid, and methyl ester were identified. Most acylglycerides (99%) constitute argan oil, whether edible or cosmetic grade (primarily triglycerides). The remaining one percent, known as unsaponifiable matter, comprises xanthophylls, triterpene alcohols, carotenes, and tocopherols (Charrouf and Guillaume, 1999; ZeinEldin et al., 2023). The primary fatty acids that constitute acylglycerides are oleic and linoleic acids, which account for 43%–49% and 29%–36%, respectively. Saturated fatty acids stearic acid and palmitic acid are present in concentrations of 4%–7% and 11%–15%, respectively (Rahmani, 2005). The high amount of unsaturated fatty acids in argan oil is the cause of some of its pharmacological characteristics. Oleic acid, a monounsaturated fatty acid, provides a wide range of health benefits (Lopez-Huertas and Health, 2010) that support the crucial qualities of argan oil. A lack of linoleic acid might result in inadequate wound healing (Galli and Calder, 2009; Almuhayawi et al., 2023a). Argan oil’s historical use for skin irritation treatment may be influenced by its high linoleic acid concentration. Due to its composition of high tocopherol concentration and unsaponifiable matter, argan oil is believed to provide several unique health advantages (Charrouf and Guillaume, 2008). Compared to olive oil, which has a tocopherol concentration of 320 mg/kg, argan oil has 620 mg/kg. Tocopherols are potent free-radical scavengers and antioxidants. Approximately 69% of the tocopherol content of argan oil is made up of gamma-tocopherol, the most effective free-radical scavenger of all tocopherols (Jiang et al., 2001; Almuhayawi et al., 2023a). It is hypothesized that a particular mix of chemicals present in the unsaponifiable matter contributes to the therapeutic effects of argan oil because sterols and tocopherols can work together synergistically (El Monfalouti et al., 2010).

Natural medicines are increasingly used worldwide, particularly in developing nations where more expensive and sophisticated treatments are not always available. This therapy has much to offer regarding maintaining and enhancing health and treating various human ailments and disorders. Since ancient times, several natural medicines have been discovered to be effective in the healing of wounds. These medications have several mechanisms of action.

Argan oil is being used more frequently in skin care. It should be emphasized that it has numerous qualities that make it a desirable target for research in the treatment of wounds. Recent research (Avsar et al., 2016) proved that this oil treated second-degree burns in rats more quickly than silver sulfadiazine, exhibiting strong TGF-1 expression and wound closure. Additionally, it has a high concentration of monounsaturated and polyunsaturated fatty acids (with an average value of more than 80%), including oleic acid (50%) and linoleic acid (30%), which have antibacterial properties (Gharby and Charrouf, 2022; Almuhayawi et al., 2023b; Hussein et al., 2023), and argan oil can shield the wound from the outside environment. However, in the present trial, no wounds developed an infection.

The main compounds identified by gas chromatography in our study are butylated hydroxytoluene, octanal, 2-(phenylmethylene)-, isopropyl myristate, oleic acid, ethyl oleate, etc., known to play a role as antimicrobials (Ribeiro Barros Cardoso et al., 2004; Villa-Rodriguez et al., 2018).

Numerous essential oils have been examined and classed as having a robust, medium, or mild antibacterial activity (Zaika, 1988). The argan essential oil under research has shown significant antibacterial effectiveness against two bacterial species in this study. The zone of inhibition more significant than 15 mm in diameter was considered a success. Generally speaking, argan essential oil significantly inhibited the growth of *P. aeruginosa* (>15 mm) and *S. aureus* (>15 mm). The existence of several active ingredients in oils, such as phenolic, alcoholic, and aldehydic contents, which have been identified as antimicrobial compounds, may be the reason for their antibacterial action (Sacchetti et al., 2005; Almuhayawi et al., 2023c; Selim et al., 2023). It has also been reported that argan oil has antibacterial activity against methicillin-resistant *Staphylococcus aureus* and *Pseudomonas aeruginosa*. Moreover, the studies indicated that treatment with argan oil alone did not yield any or had very low antibacterial activity against methicillin-resistant *Staphylococcus aureus* and *Pseudomonas aeruginosa*, respectively (Naher et al., 2014; Elnosary et al., 2022). A study by Stojkovic et al. (2013) found that the naturally occurring compounds, caffeic acid, p-coumaric acid, and rutin, present in argan oil could inhibit *Staphylococcus aureus* growth. The available research suggests that the compounds present in argan oil and other crude extracts of the argan tree can be considered for the further evaluation of their antibacterial activity against *Cutibacterium acnes* (*C. acnes)* and *Prevotella intermedia (P. intermedia)*.

According to MIC results, all vital argan essential oils inhibited *P. aeruginosa* at concentrations over 0.3 mg/mL and *S. aureus* at concentrations above 0.5 mg/mL. Lemon grass had similar MIC values (0.25 mL/100 mL) against *P. aeruginosa* and *S. aureus* (Adegoke and Odesola, 1996; Elnosary et al., 2023). However, Stojkovic et al. (2013) investigated the MIC values for argan oils against *C. acnes* and *P. intermedia* and discovered that concentrations 500 and 12,500 ug/mL/were microbiocidal, which is consistent with our findings.

Data from the disc diffusion technique and MIC show that argan essential oil suppressed *P. aeruginosa* (Gram-negative) and *S. aureus* (Gram-positive). Gram-positive appeared to be more sensitive to these oils. It was discovered that Gram-positive bacteria were more vulnerable than Gram-negative bacteria. *Staphylococcus aureus* (*S. aureus*) is a Gram-positive bacterium, characterized by its thick peptidoglycan layer in the cell wall. This might be because Gram-positive bacteria’s cell walls are less complicated and lack a natural filter effect against giant molecules because of the tiny pores in their cell envelope (Moreira et al., 2005). The reported resistance of the studied organisms may be caused by other genetic variables or by the permeability of the cell membrane.

## 4 Materials and methods

### 4.1 Plant material

Argan seeds were collected from Sakaka, Saudi Arabia’s Al-Jouf market. The seeds were crushed and combined with standard pellets.

### 4.2 Oil extraction

Argan seeds were cold-pressed. The following step involved grinding and compressing the seeds (2 kg) using a conical screw rotation. The extracted oil was forced through a perforated tube, and the meal was expelled via a calibrated aperture to get 100 g of concentrated extract. After that, a centrifuge was used to separate the oil from the plant debris (15 min), filtered, and kept frozen at a temperature of 20°C.

### 4.3 Extract preparation and purification

For additional research, potent extracts with a substantial inhibition zone were chosen. The most potent plant seeds’ powders were dissolved in ethanol. The ethanolic extract was centrifuged at 4,300 rpm for 15 min to remove the cellular components. The pellet was refined after three further extractions. Six referenced standard Oxoid antibiotic discs (tetracycline 10 g, streptomycin 10 g, chloramphenicol 30 g, nitrofurantoin 300 g, vancomycin 30 g, and rifampicin 5 g) were used to compare the antibacterial activity of fraction.

### 4.4 GC/MS analysis

Thermo Scientific’s Trace GC-TSQ mass spectrometer and TG-5MS direct capillary column (30 m × 0.25 mm x 0.25 m film thickness) were used to analyze the chemical composition of argan oil. The temperature of the column oven was initially maintained at 50°C, then increased by five °C/min to 250°C and held for 2 min, and then, increased to the ultimate temperature of 300°C by 30°C/min and kept for 2 min. Helium was used as the carrier gas, with a constant flow rate of 1 mL/min, and temperatures of the injector and MS transfer line were maintained at 270 and 260°C, respectively. Autosampler AS1300 paired with GC in split mode automatically injected diluted samples of 1 L with a solvent delay of 4 min. m/z 50–650 EI mass spectra were gathered in full-scan mode at 70 eV ionization voltages. The ion source temperature was fixed at 200 °C by comparing the mass spectra of the components to those in the WILEY 09 and NIST 14 mass spectral databases (Abd El-Kareem et al., 2016).

### 4.5 Molecular docking study

A molecular modeling study was performed to predict the interaction mechanism between two interacting chemical entities (receptor sites and inhibitors). In this study, we utilized computational docking simulation to predict 3D binding modes using the online SwissDock server (Grosdidier et al., 2011).

### 4.6 Evaluation of the ADME, pharmacokinetics, and medicinal chemistry friendliness of the isolated compounds

The term “ADME” is the four-letter acronym for absorption, distribution, metabolism, and excretion, describing pharmacokinetics. The term “ADME” refers to the internal processes that explain how a drug enters the body, gets processed by it, and determines how much of a drug is harmful to an organism. ADMET properties play an important role as they account for the failure of 60% of drug molecules during the drug development process.

The goals of clinical pharmacokinetics include enhancing the efficacy and decreasing the toxicity of a patient’s drug therapy. The development of strong correlations between drug concentrations and their pharmacologic responses has enabled clinicians to apply pharmacokinetic principles to actual patient situations. The pharmacokinetic studies were to determine bioavailability. Bioavailability, which refers to the extent and rate at which a drug enters the circulatory system, is a measure of its access to its target and site of action.

Using the open-source OSIRIS Property Explorer, we evaluated the toxicity risks, drug-relevant properties, drug-likeness, and drug score for isolated compounds (https://www.organic-chemistry.org/prog/).

SwissADME is an accessible online web server that we used to predict the essential parameters for our isolated phytochemicals to be utilized as an effective drug candidate. A drug molecule must be delivered to its action site at sufficient concentration to induce the desired biological response and stay there for a sufficient time. Accordingly, medicinal chemists apply different computer models to predict different physicochemical properties of drug molecules. The Online SwissADME web browser (http://www.swissadme.ch) was used to submit the isolated phytochemicals to be estimated for medicinal chemistry friendliness, drug-likeness, ADME, and pharmacokinetics (Daina et al., 2017).

### 4.7 Bacterial strains

The *P. aeruginosa* and *S. aureus* strains were from the bacteria collection of the Department of Clinical Laboratory Sciences, College of Applied Medical Sciences, Jouf University, Sakaka, Saudi Arabia. Antibiotic resistance was determined by the width of the inhibitory zones surrounding the antibiotic discs after 48 h of incubation at 37°C. Oxoid standard antibiotic discs were utilized. After 24 h of incubation at 37°C, the diameter of clear zones around the antibiotic discs showing bacterial growth inhibition was determined. According to the manufacturer’s specifications, bacterial isolates were categorized as “resistant,” “intermediate,” or “sensitive.”

### 4.8 Antibacterial activity screening

Argan essential oil was tested for its antibacterial properties against the research strains of *S. aureus* and *P. aeruginosa*. The Bauer et al. (1966) single-disc approach was applied. Several bacterial species were cultured (108 cells/mL) in a nutrient broth and incubated at 37°C for 24 h. After that, Müeller–Hinton agar was added to Petri dishes where they were placed (Oxid Products, UK). After being embedded in 20 L of the oil lipid-soluble extracts, sterile discs of 5-mm diameter were introduced to the various bacterial cultures. After 24 h, the halo diameter surrounding the discs was used to determine the antibacterial activity of argan seed oil against the tested microorganisms. Every experiment was carried out twice. The negative control used discs without oil embedded in 50% methanol; in this instance, no antibacterial action was seen.

### 4.9 Determination of the MIC, MBC, and tolerance ratio

The MIC was identified as the most negligible concentration of an antibiotic that inhibited the development of visible bacterial growth in broth (Coello et al., 2020). The MIC was identified by the broth dilution method. The authors added serial dilutions of 0.1–1 mg of essential oil to Müller–Hinton broth. They also added a bacterial suspension of 1 mL with approximately 10^5^:5×10^5^ cells of *S. aureus* and *P. aeruginosa* to each dilution. They checked the bacterial growth following overnight incubation at 37°C. The MBC was identified as the minor concentration of antibiotic that destroys 99.9% of the organism. Since the organisms are quantitatively subcultured from MIC tubes on an antibiotic-free agar medium to determine that the minimal concentration was no viable organism in the culture, it is frequently an extension of the MIC (Collins, 1967). The authors estimated the degree of tolerance from MBC/MIC readings (El and Housseini, 2015).

### 4.10 Scanning electron microscopy

*S. aureus* and *P. aeruginosa* solutions (10 (ZeinEldin et al., 2023) CFU/mL) with argan oil (at approximately MIC) were spotted onto polylysine-coated glass slides for SEM. The next step was to fix each slide by soaking it in 1 mL of 2.5 percent glutaraldehyde and 2 percent paraformaldehyde in 0.1 M sodium cacodylate buffer (pH 7.2) for 12 h, followed by post-fixation in 1% OsO4 for 2 h. The fixed samples were dehydrated in a gradient of ethanol (70, 80, 90, and 100°GL) before being critical point dried in CO2 (BALTEC CPD 030 Critical Point Dryer). The slides were then secured with tape to stubs, coated with gold using the BALTEC SDC 050 Sputter Coater, and examined using the FEI Quanta 200 SEM. All reagents were obtained from Sigma-Aldrich, USA.

### 4.11 Statistical analysis

All the data were entered and evaluated by exhausting the Statistical Package for Social Science version 24 (IBM Corp., Armonk, NY, USA). *p*-value <0.05 indicates statistical significance.

## 5 Conclusion

The present study confirmed the antimicrobial properties of argan oil that showed significant growth inhibition for *S. aureus* and *P. aeruginosa* isolated from the Department of Clinical Laboratory Sciences, College of Applied Medical Sciences, Jouf University, Sakaka, Saudi Arabia. The problem relates to the emergence of strains that possess multiple resistance to a range of antibiotics, thereby making them difficult to treat. GC/MS studies on the composition of argan oil showed that the occurring compounds, including butylated hydroxytoluene, oleic acid, ethyl oleate, 10-octadecenoic acid, and methyl ester, could impede the growth and proliferation of *S. aureus*. Considering the available data, it may be worthwhile to further investigate the potential antibacterial activity of the compounds present in argan oil and other crude extracts of the argan tree. In addition to its antibacterial properties, argan oil is beneficial as a lipid carrier in nanoemulsions and for the topical administration of other drugs by increasing skin permeability. The encouraging results indicate that argan oil might be exploited as a natural antibiotic for the treatment of several infectious diseases caused by these two germs and could be useful in understanding the relations between traditional cures and current medicines.

## Data Availability

The original contributions presented in the study are included in the article/Supplementary Material; further inquiries can be directed to the corresponding authors.
