# Supplementary material for: In vitro and in silico biopotentials of phytochemical compositions and antistaphylococcal and antipseudomonal activities of volatile compounds of Argania spinosa (L.) seed oil
Source: Front Bioeng Biotechnol. 2024 Mar 13;12:1348344. doi: 10.3389/fbioe.2024.1348344 (PMC10965789; doi:10.3389/fbioe.2024.1348344)
Supplement: Supplementary file 1 [file DataSheet1.docx]

| 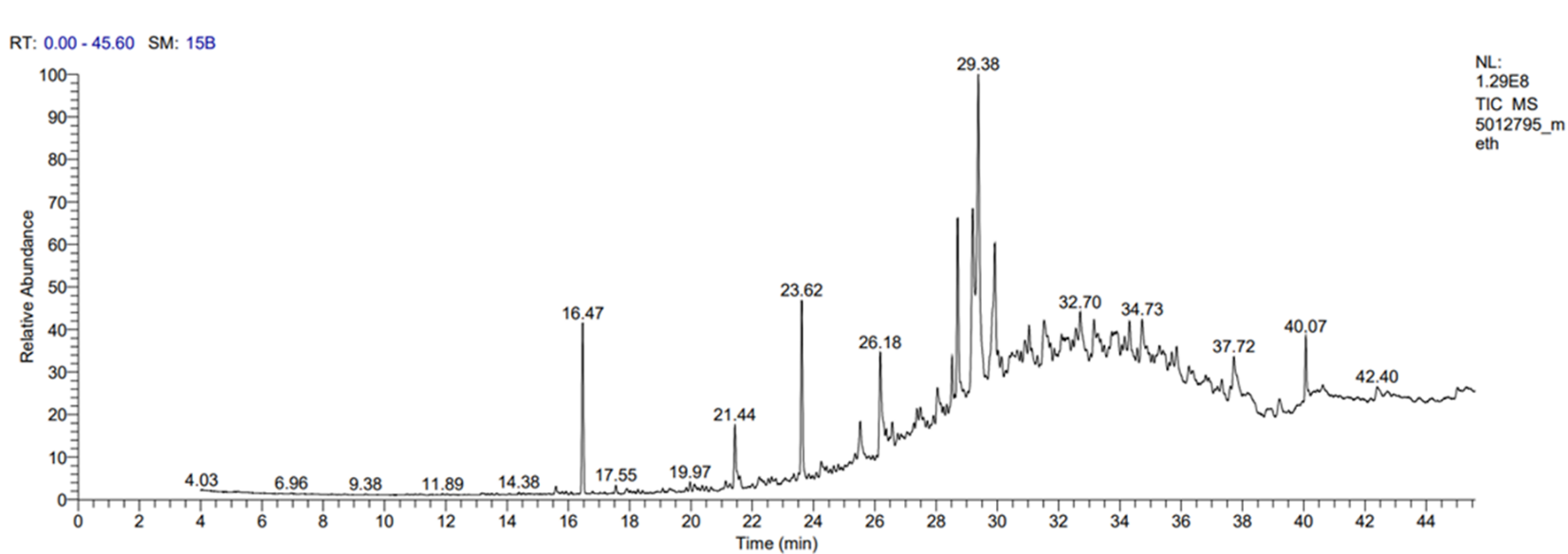  **a** |
| --- |
| 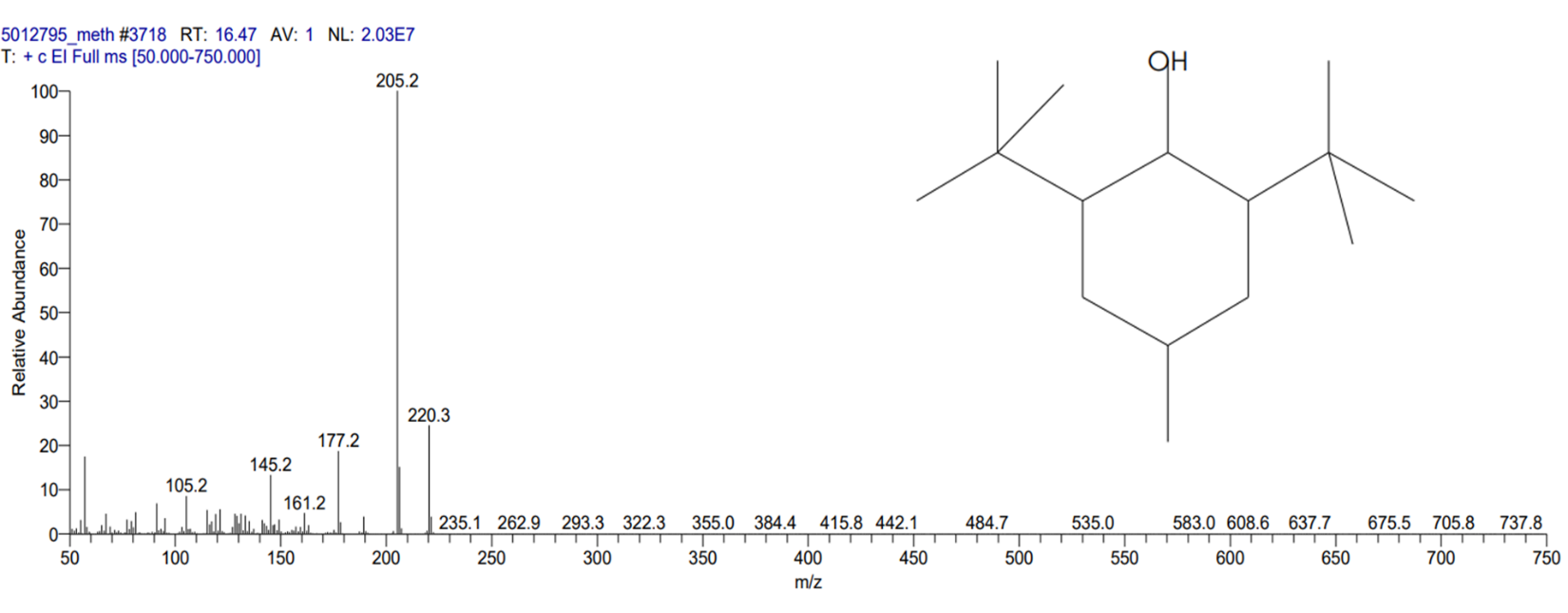  **b** |
| 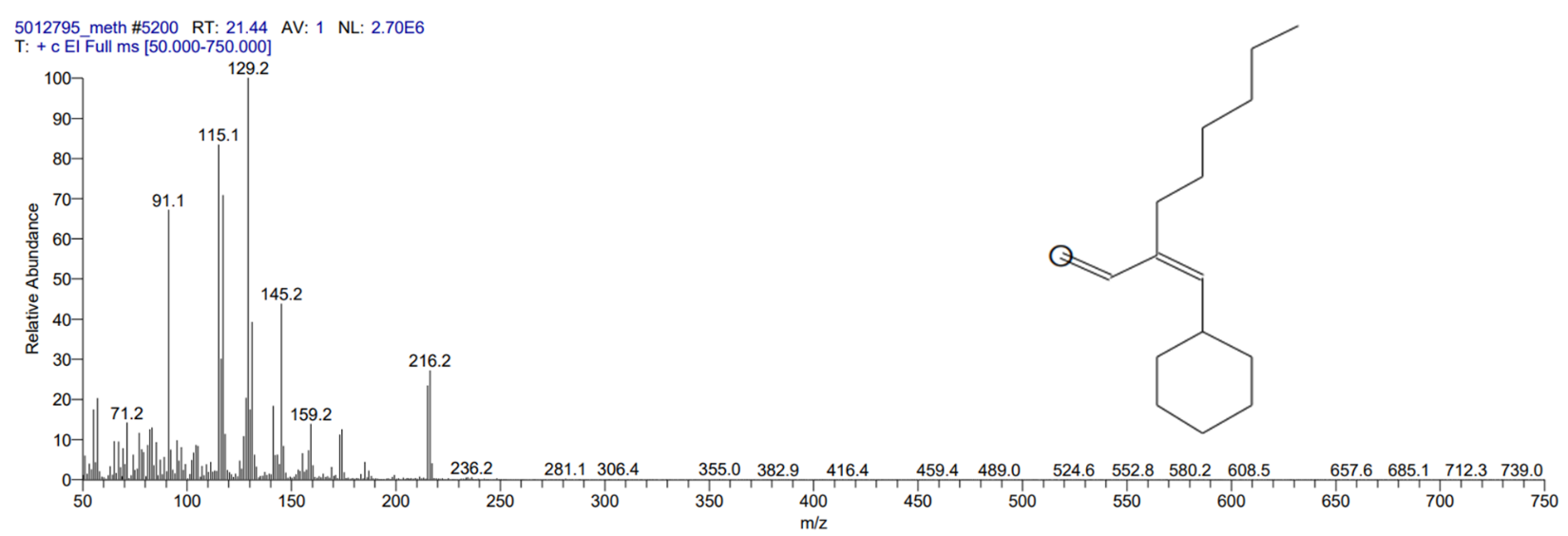  **c** |
| 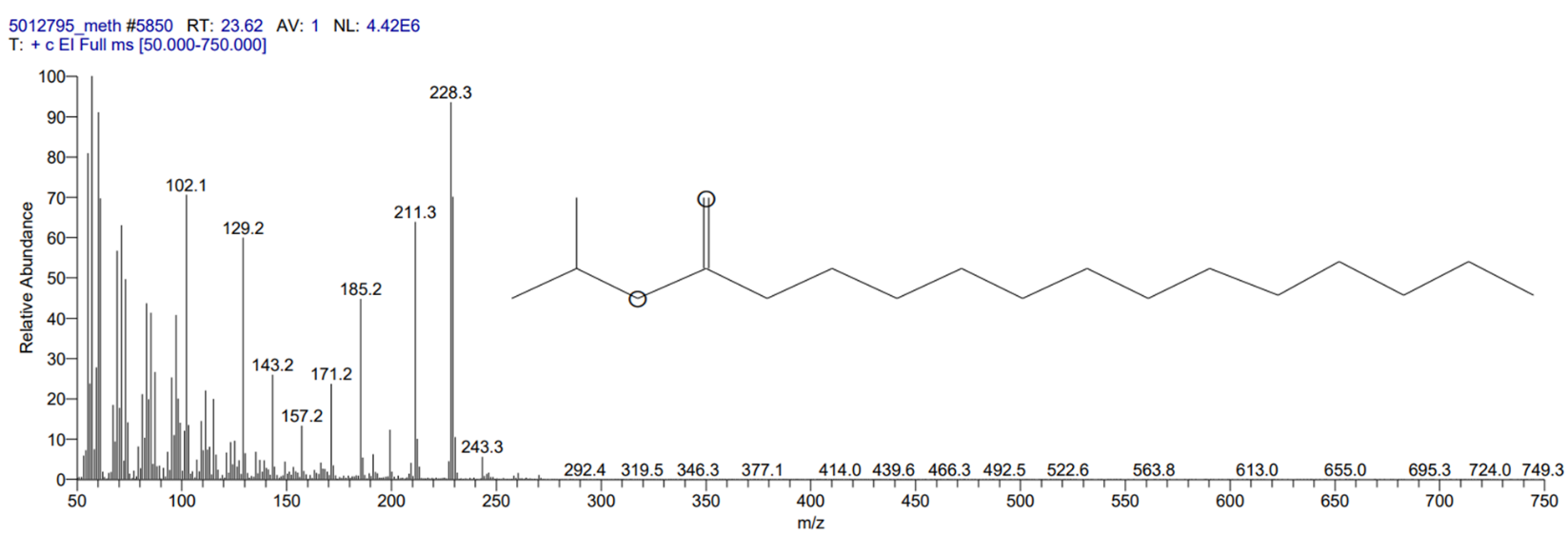  **d** |
| 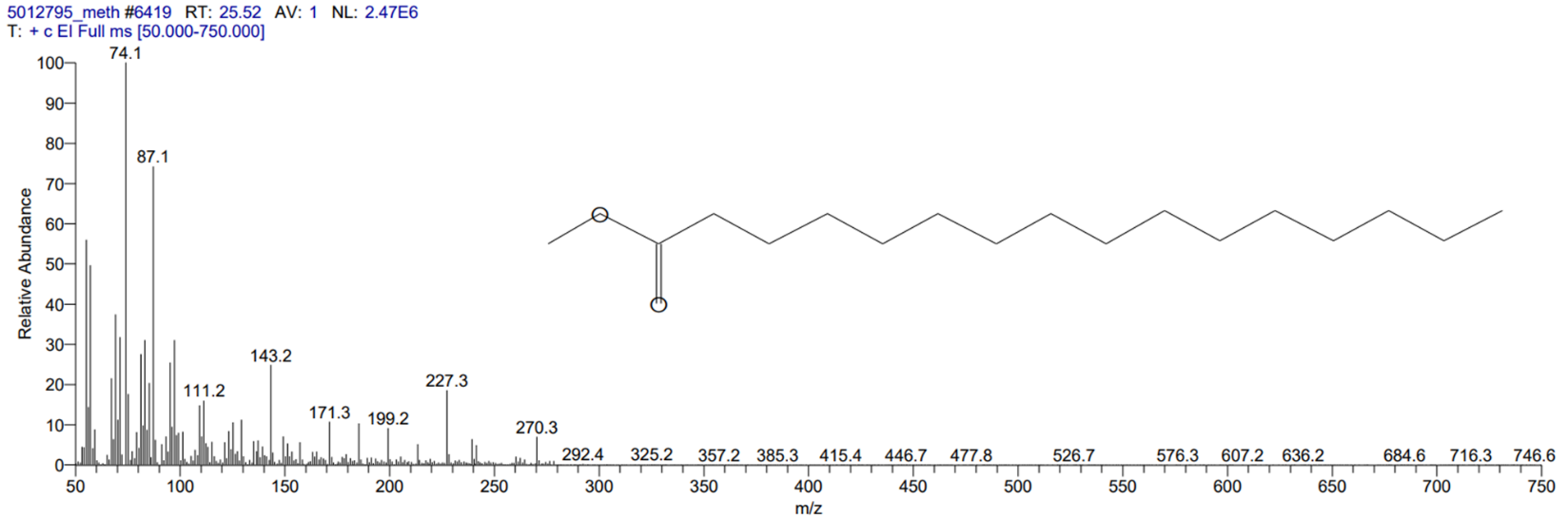  **e** |
| 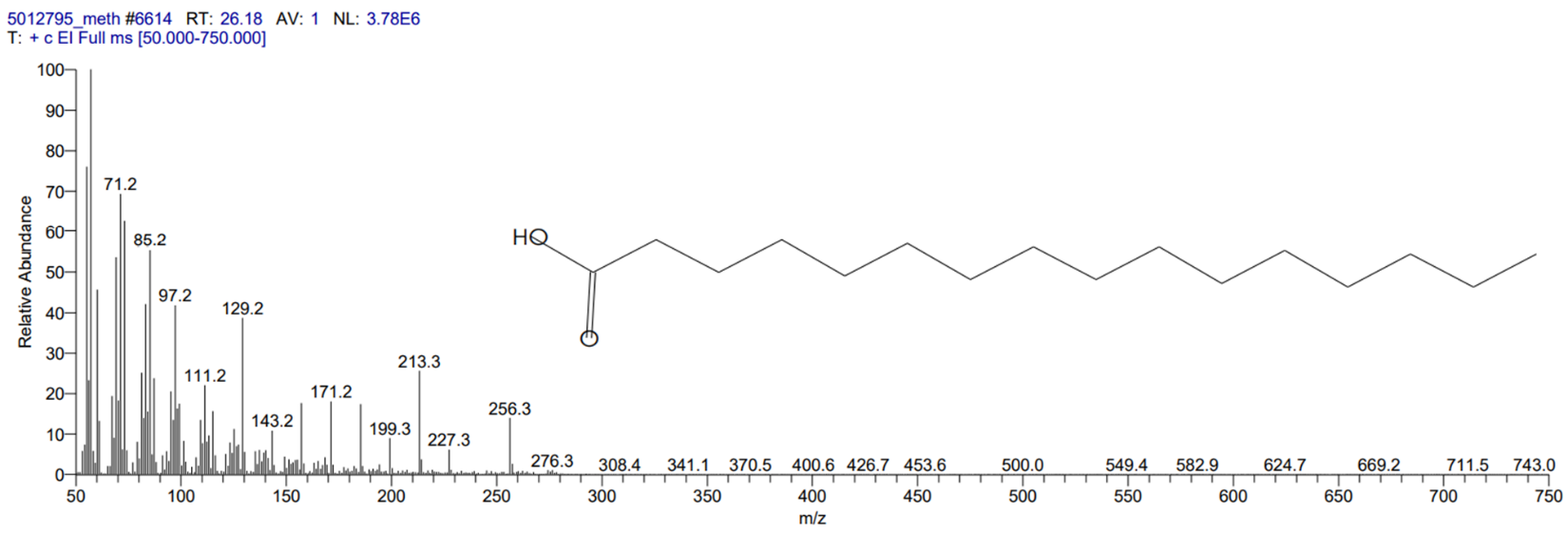  **f** |
| 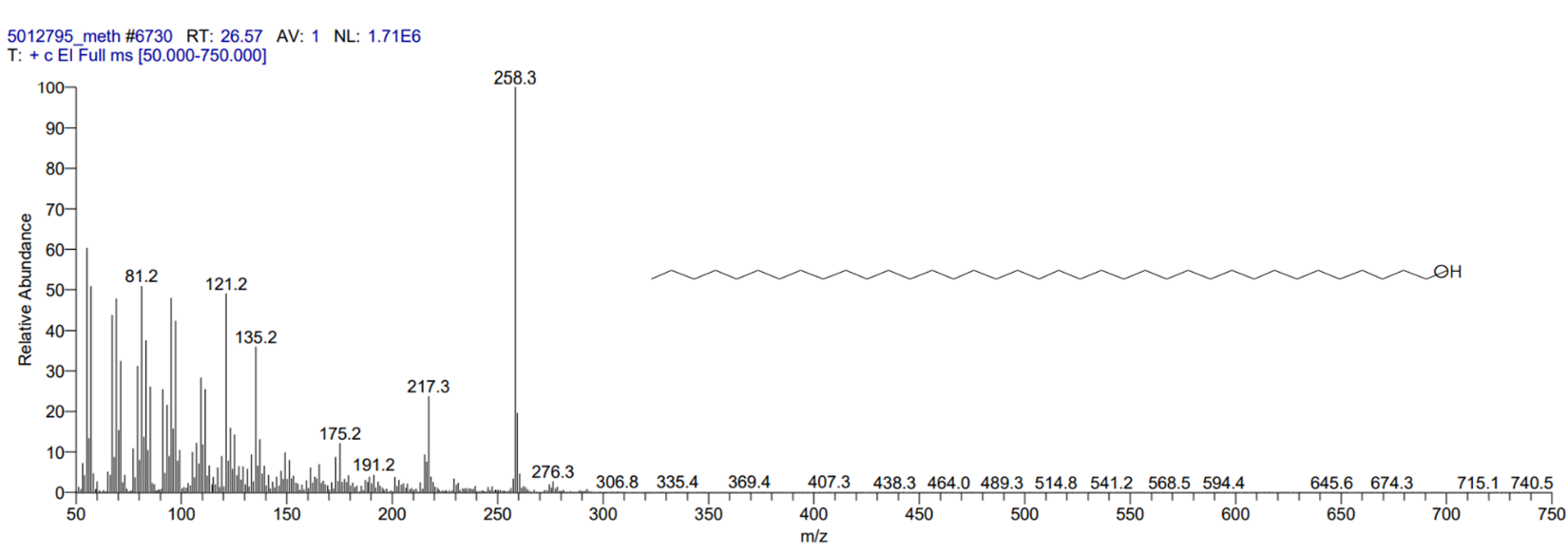  **g** |
| 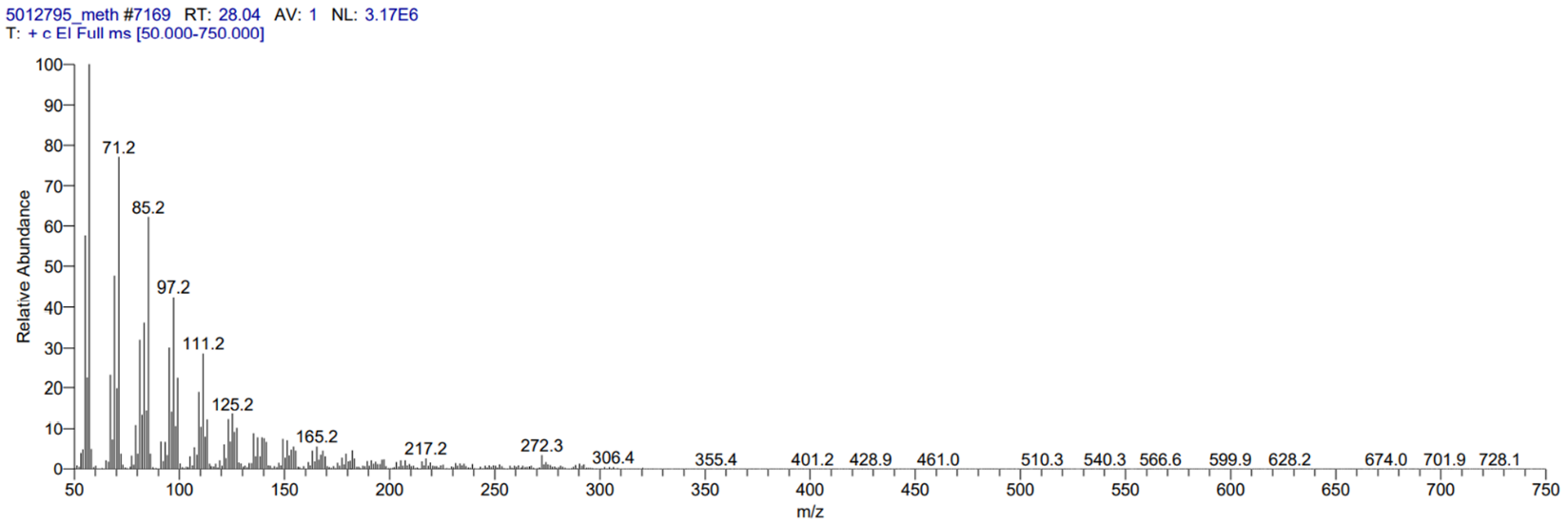  **h** |
| 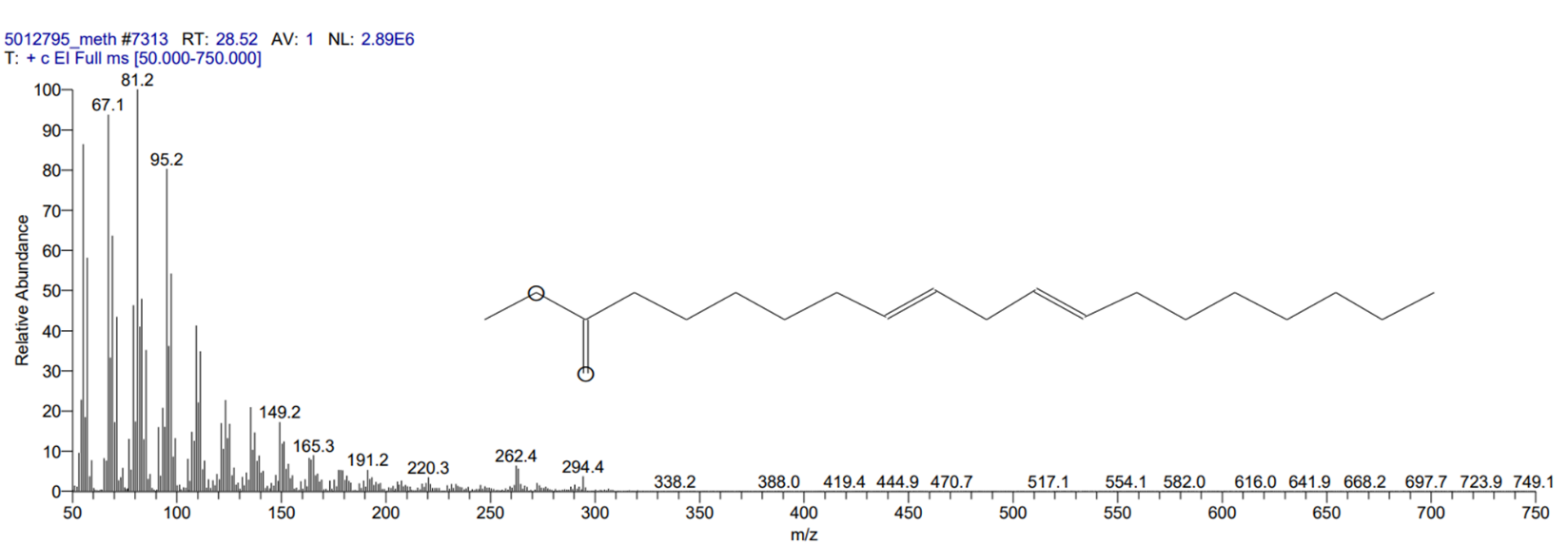  **i** |
| 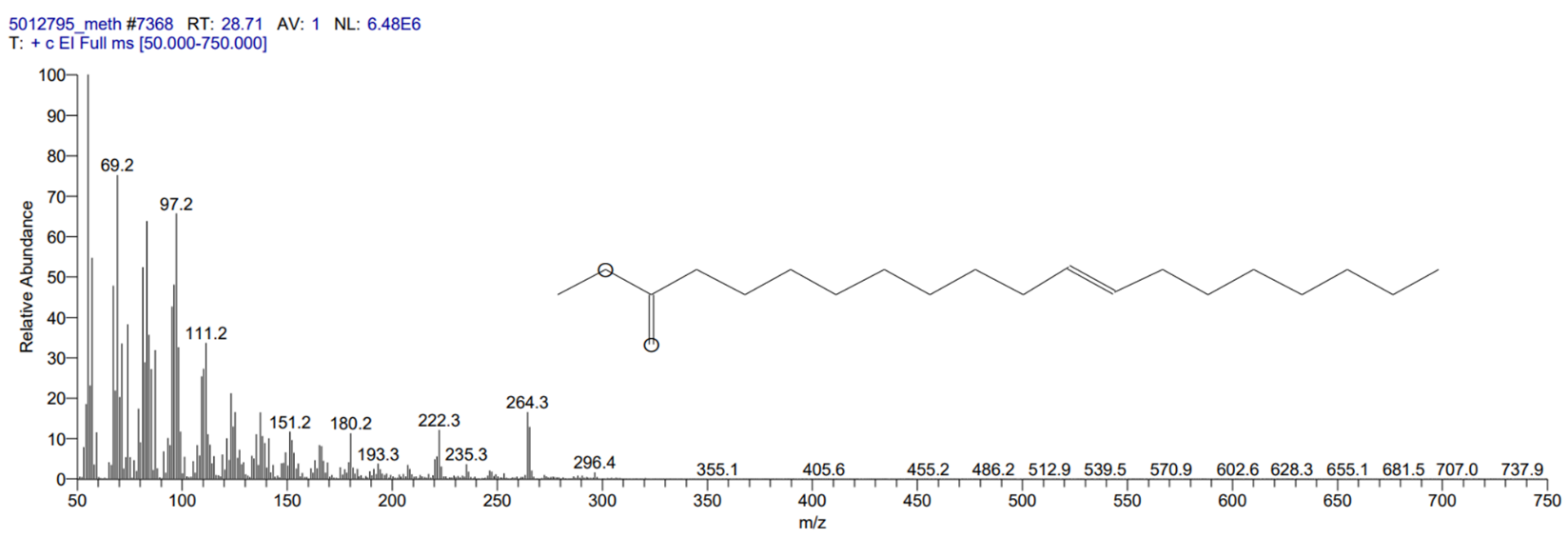  **j** |
| 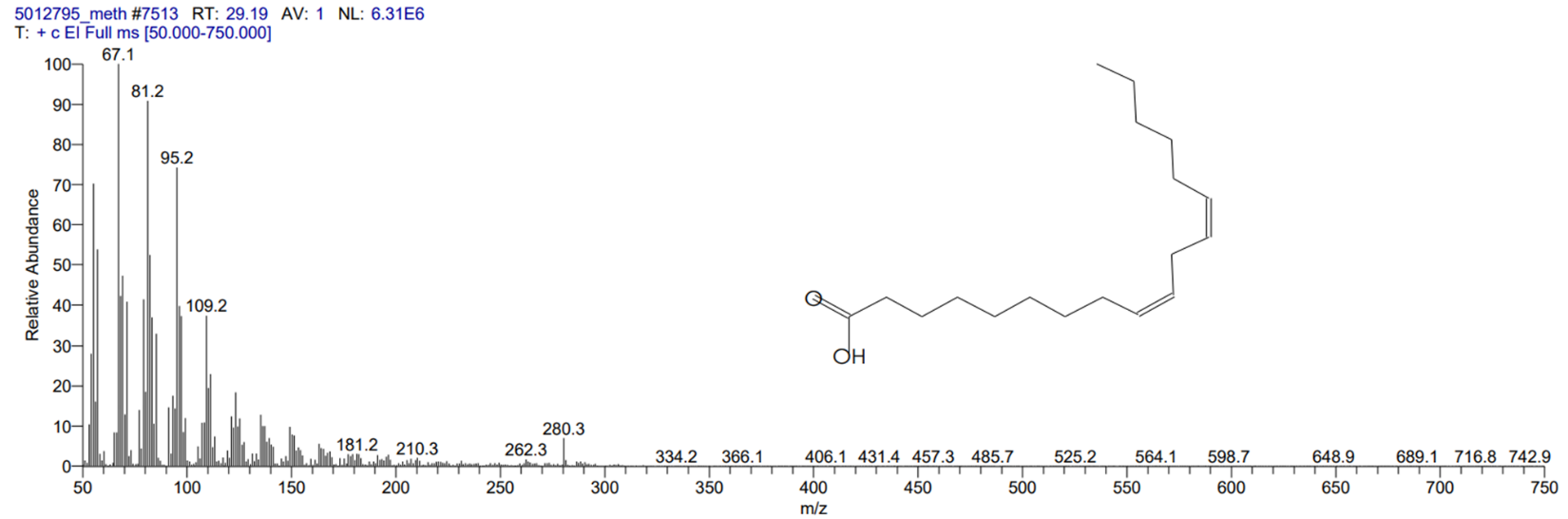  **K** |
| 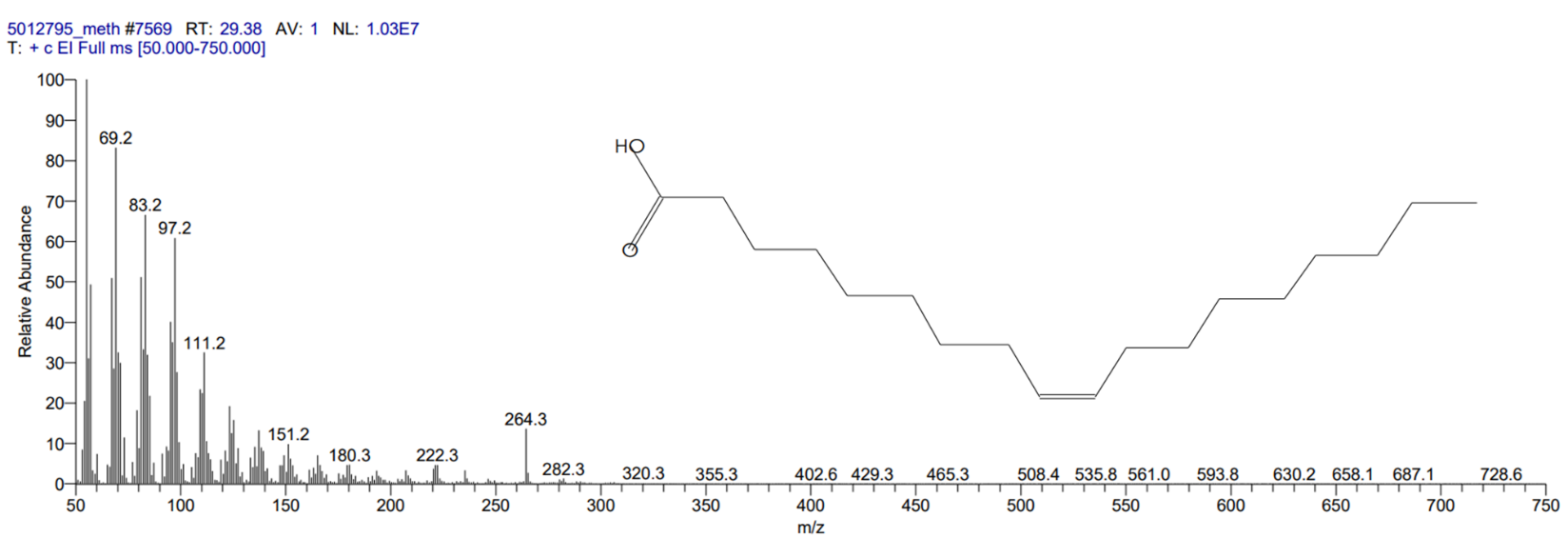  **l** |
| 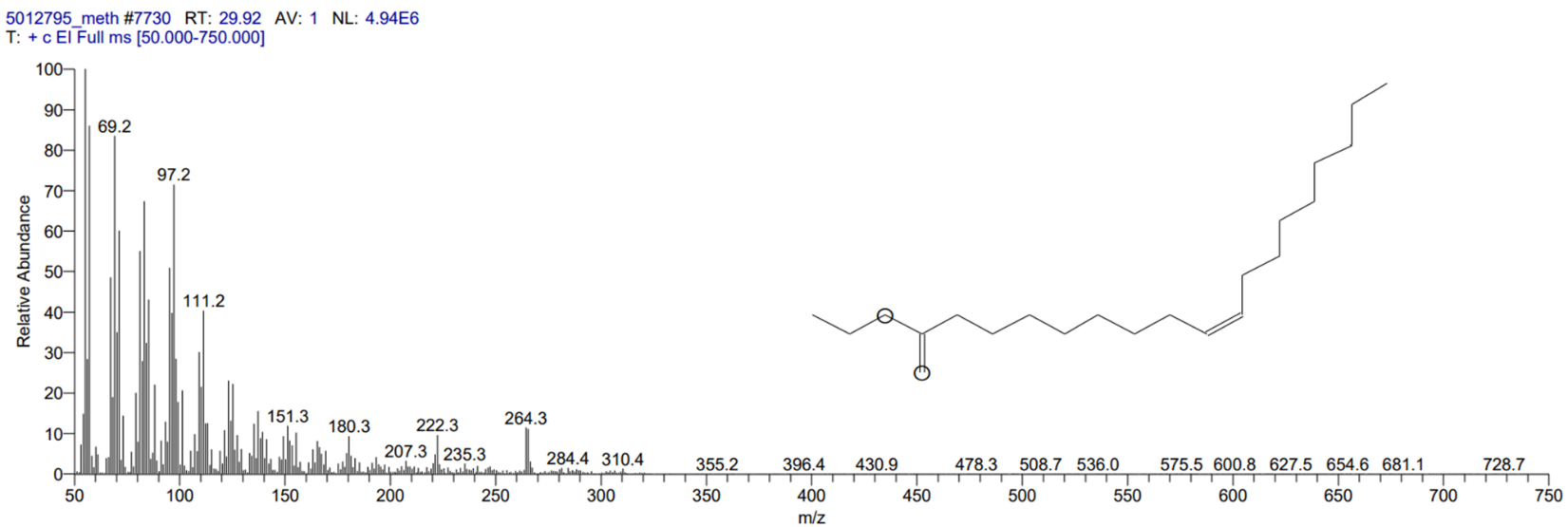  **m** |
| 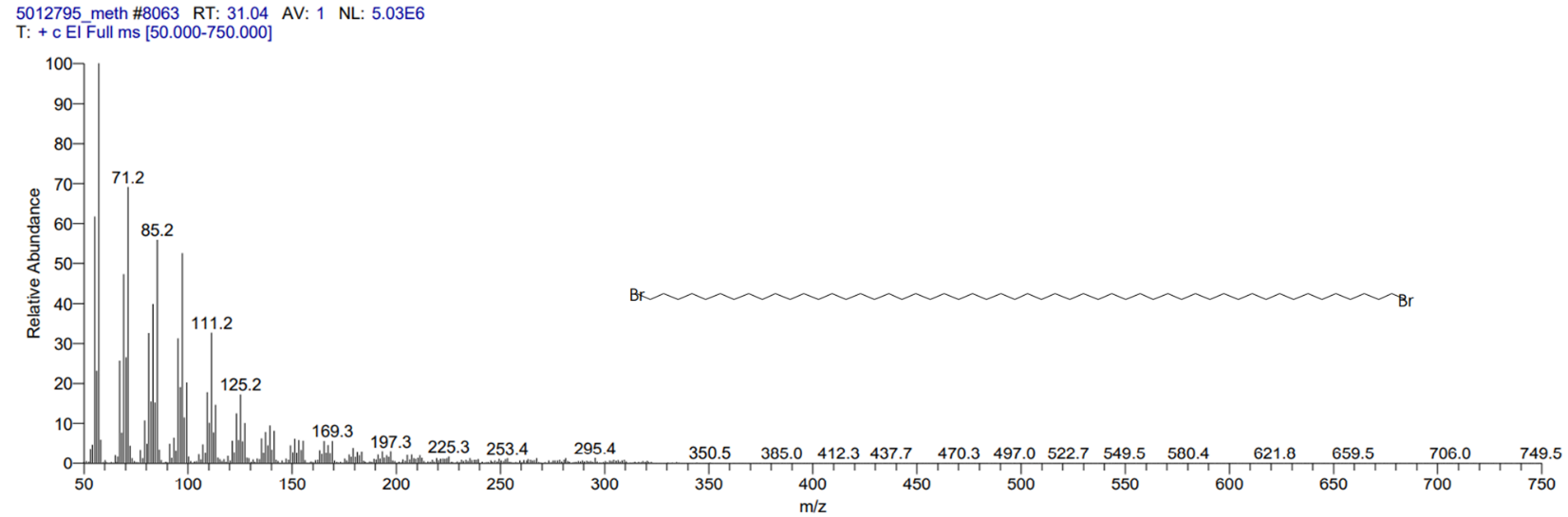  **n** |
| 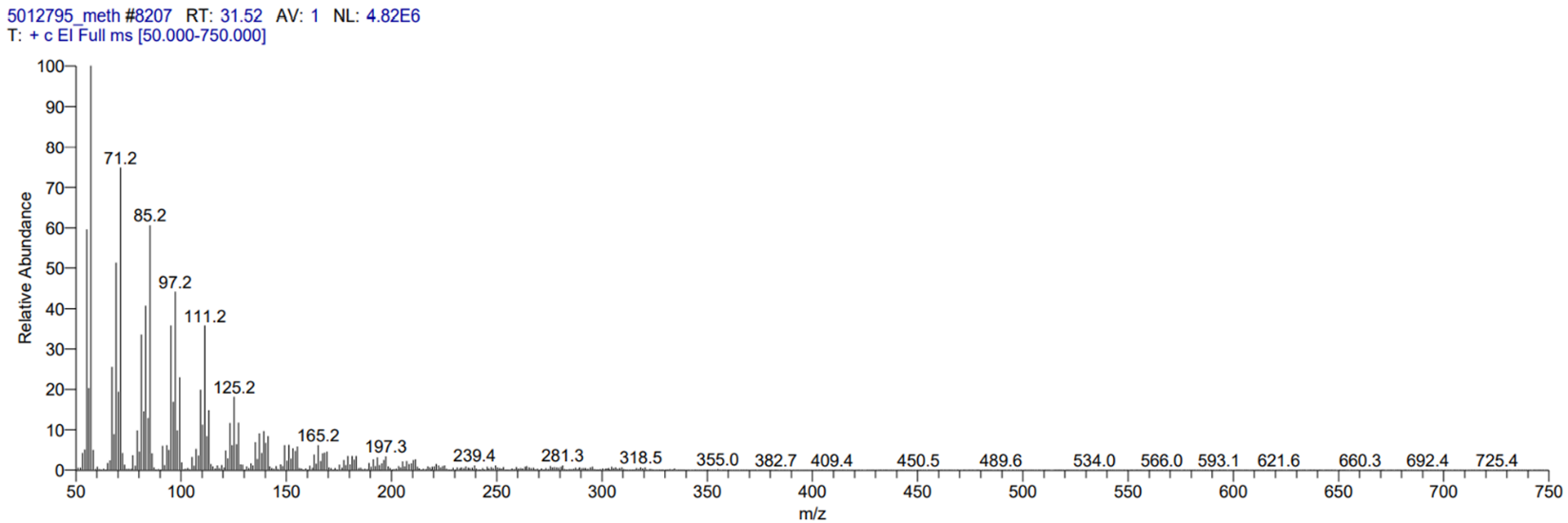  **o** |
| 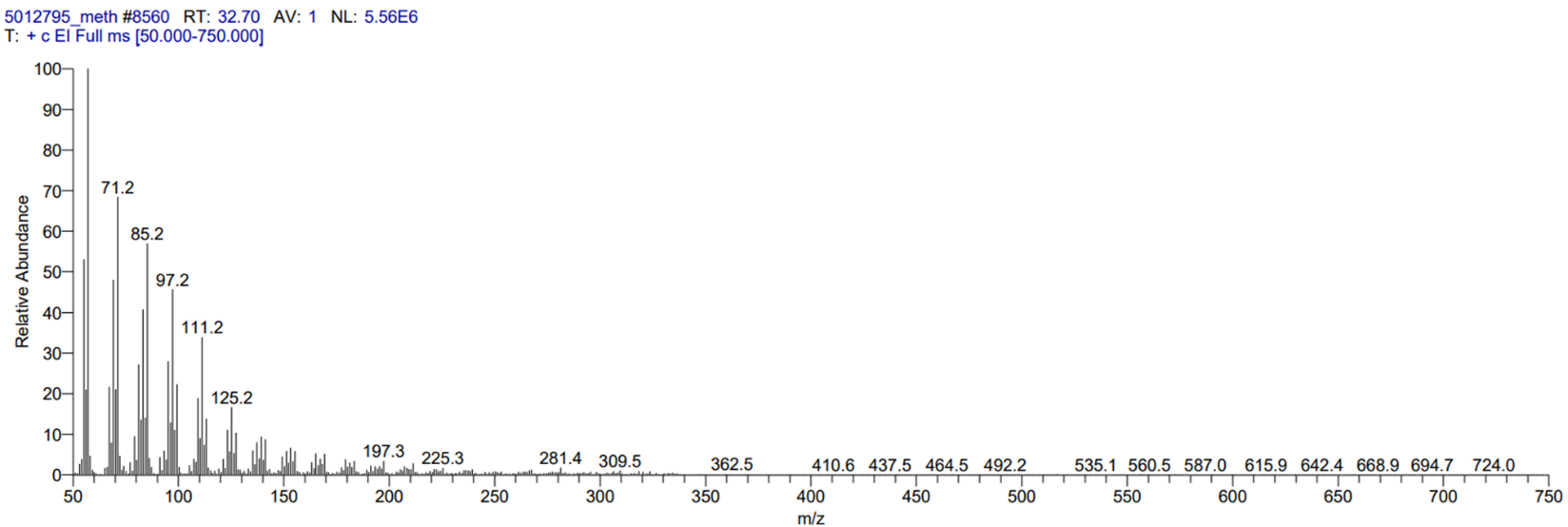  **p** |
| 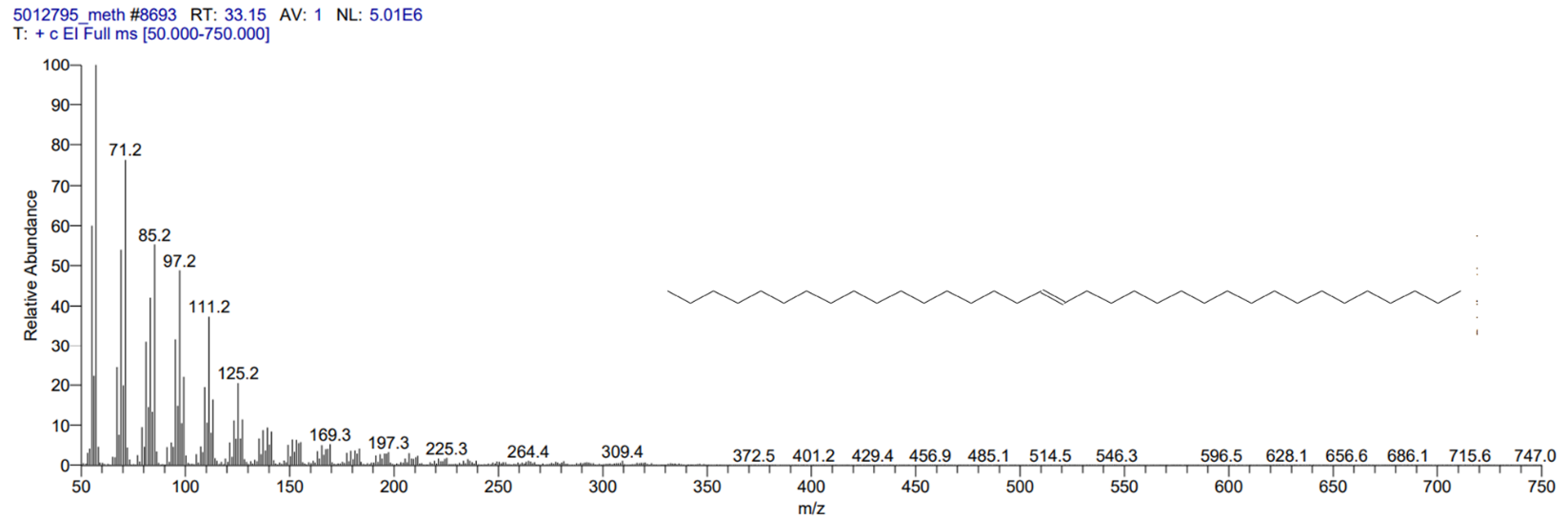  **q** |
| 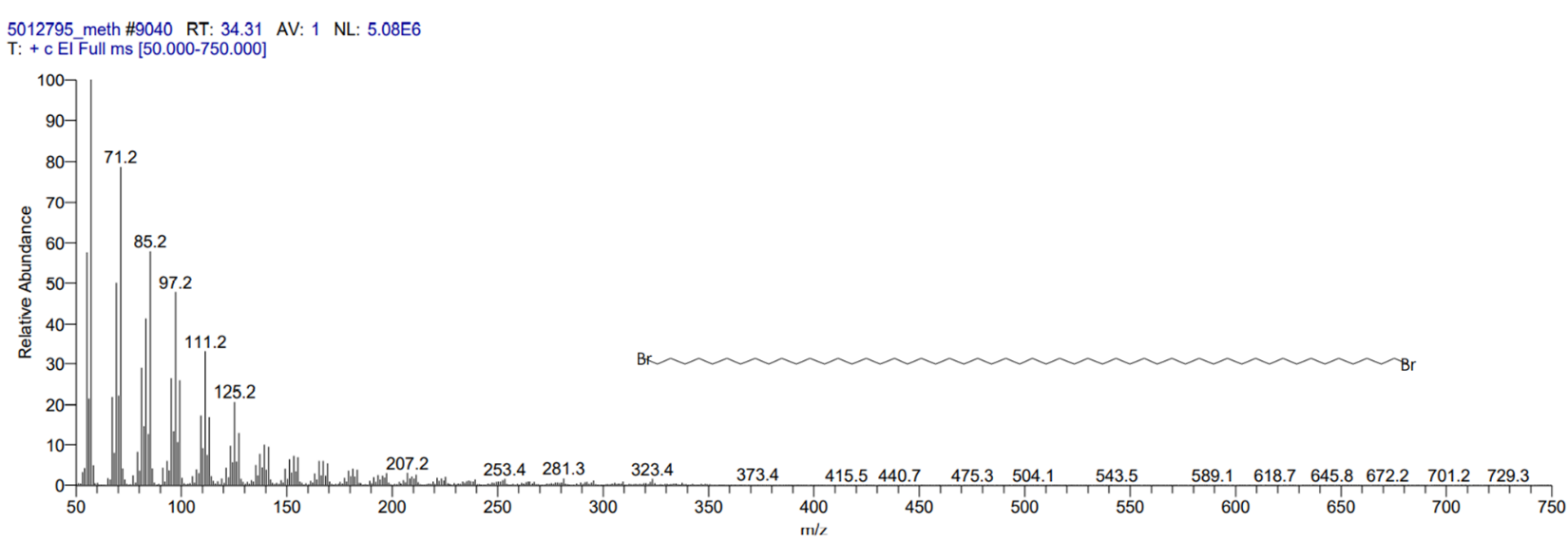  **r** |
| 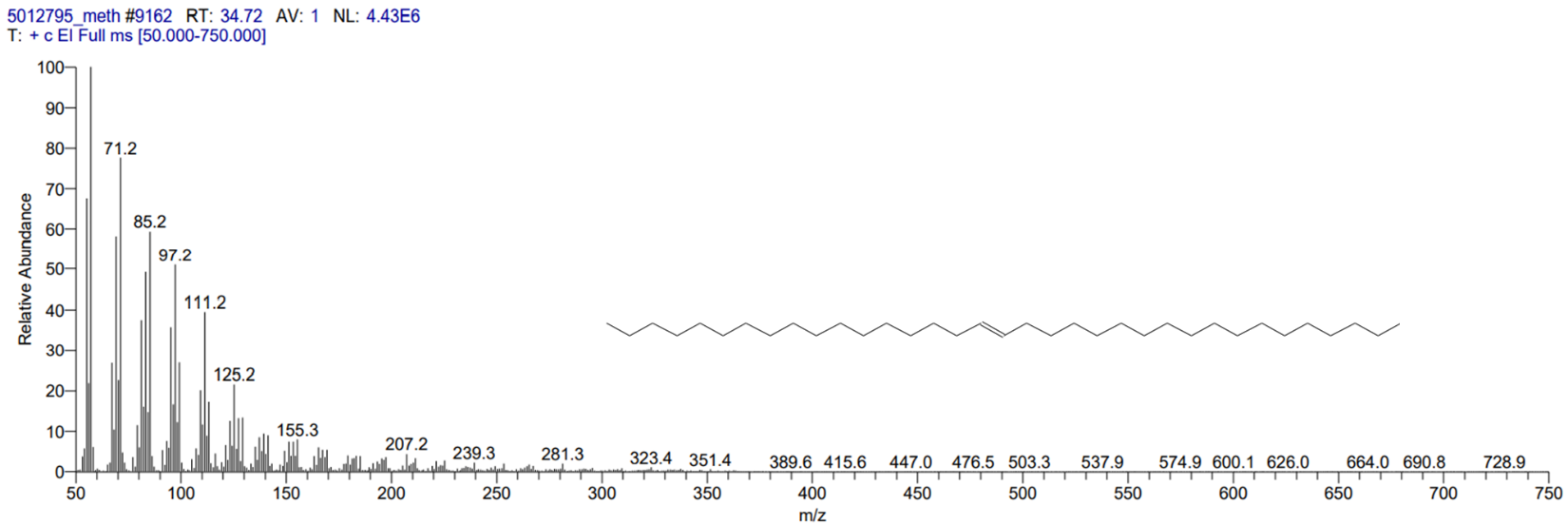  **s** |
| 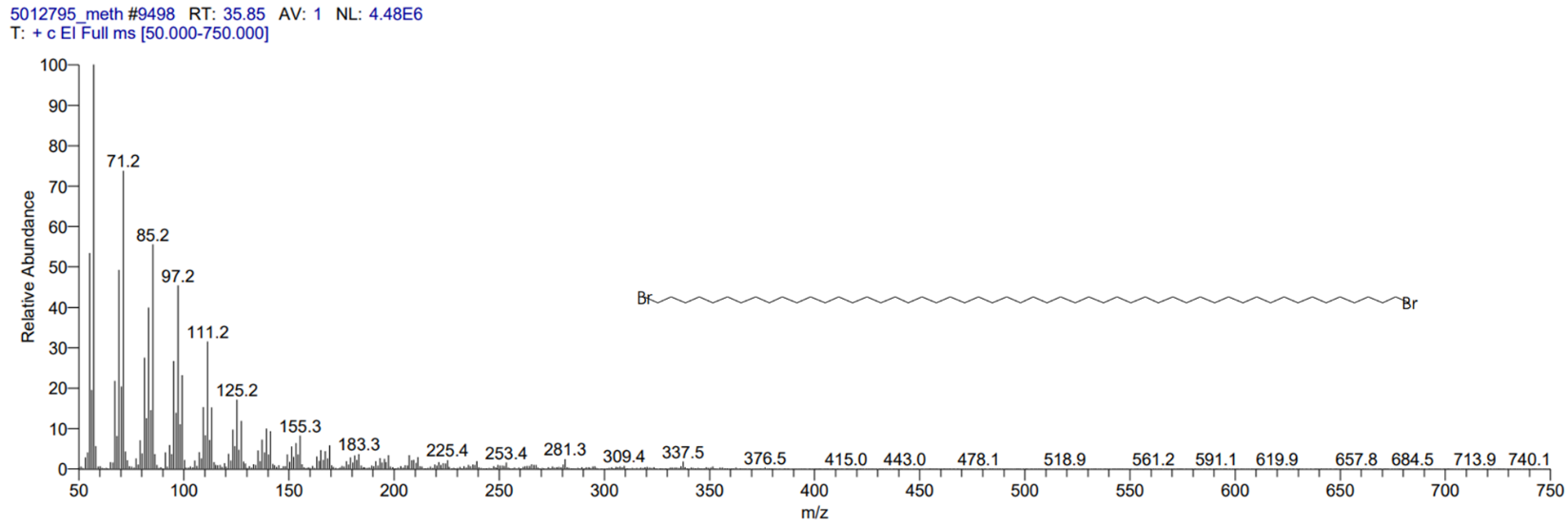  **t** |
| 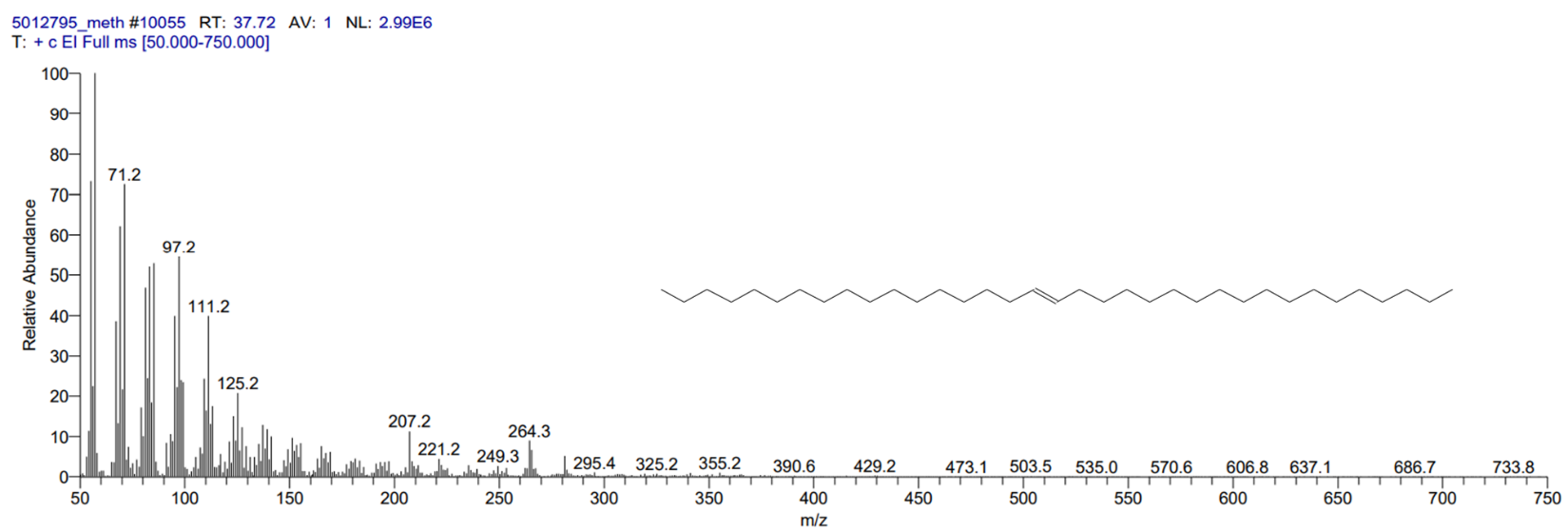  **u** |
| 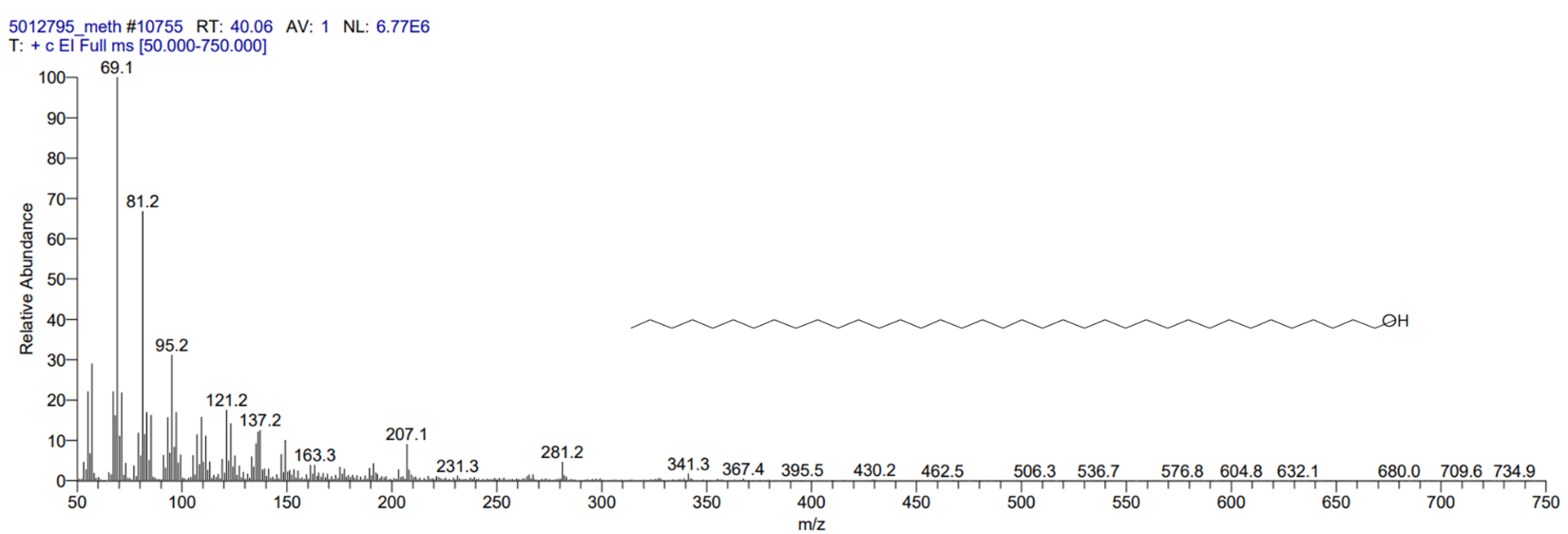  **v** |

**Figure S1.** The total ion chromatogram of (a) the Argan oil and the mass spectrum of (b) Butylated Hydroxytoluene, (c) Octanal, 2-(phenylmethylene)-, (d) Isopropyl myristate, (e) Palmitic Acid methyl ester, (f) Hexadecanoic acid, (g) 1-Heptatriacotanol, (h) 14-á-H-Pregna, (i) 7,10- Hexadecanoic acid, methyl ester, (j) 10-Octadecenoic acid, methyl ester, (k) 9,12-Octadecadienoic acid (Z,Z)-, (l) Oleic Acid, (m) Ethyl Oleate, (n) Tetrapentacontane, 1,54-dibromo-, (o) 14-á-H-Pregna, (p) 14-á-H-Pregna, (q) 17-Pentatriacontene, (r) Tetrapentacontane, 1,54-dibromo-, (s) 17-Pentatriacontene, (t) Tetrapentacontane, 1,54-dibromo-, (u) 17-Pentatriacontene, and (v) 1-Heptatriacotano.
